# Supplementary material for: Δccr5 Genotype Is Associated with Mild Form of Nephropathia Epidemica
Source: Viruses. 2019 Jul 23;11(7):675. doi: 10.3390/v11070675 (PMC6669606; doi:10.3390/v11070675)
Supplement: Supplementary file 1 [file viruses-11-00675-s001.zip › viruses-523619-supplementary - 2 - KK - 1.docx]

Supplemental data:

Table 1. Clinical characteristics and laboratory data on all NE cases.

| Variable | Value |
| --- | --- |
| Age (years) | 37.50 [29.75, 51.50] n=98 |
| Hospitalization (days) | 11.00 [8.00, 12.00] n=96 |
| Fever (days) | 6.00 [4.00, 7.00] n=54 |
| Back pain (days) | 8.00 [4.00, 10.00] n=55 |
| Oliguria (days) | 2.00 [0.00, 3.00] n=98 |
| Urea (mg/dL) | 7.10 [4.70, 12.60] n=93 |
| Creatinin (µmol/dL) | 131.00 [103.00, 189.00] n=93 |
| Thrombocytes (cells/μl) | 92.00 [67.00, 157.00] n=98 |
| White blood cells (cells/μl) | 9.30 [6.20, 13.65] n=55 |
| Anti-hantavirus IgM (negative/positive (%)) | 7/91 |
| Anti-hantavirus IgG (negative/positive (%)) | 5/93 |
| Gender (female/male (%)) | 18/80 (18.4/81.6) n=98 |

Continuous variables are presented with their respective median, first (Q1) and third (Q3) quartiles (median [Q1, Q3], total n). Categorical variables are presented with cross tables and percentages (negative / positive (percentage), total n).

Table 2. Serum cytokine, chemokine and MMP levels in functional CCR5 homozygous NE cases and controls (median [Q1, Q3]).

| Variable | Control | wtCCR5 homozygous NE | p | Adjusted p |
| --- | --- | --- | --- | --- |
| IL1α | 0.80 [0.18, 1.33] n=27 | 0.14 [0.10, 0.20] n=80 | <0.001 | <0.001 |
| IL2RA | 27.52 [13.53, 40.51] n=27 | 75.44 [42.21, 142.03] n=80 | <0.001 | <0.001 |
| IL3 | 67.64 [33.36, 101.21] n=27 | 84.69 [61.74, 169.87] n=80 | 0.023 | 0.048 |
| IL8 | 11.40 [8.60, 32.58] n=25 | 44.00 [24.00, 89.95] n=54 | 0.001 | 0.002 |
| IL10 | 6.60 [3.52, 10.45] n=27 | 16.34 [9.21, 43.74] n=80 | <0.001 | <0.001 |
| IL11 | 1.49 [0.87, 2.27] n=27 | 5.40 [2.42, 20.07] n=54 | <0.001 | <0.001 |
| IL12B | 70.80 [36.59, 154.07] n=27 | 161.56 [97.07, 290.62] n=80 | 0.001 | 0.004 |
| IL16 | 105.65 [72.40, 141.87] n=27 | 180.11 [124.42, 289.76] n=80 | <0.001 | <0.001 |
| IL18 | 5.76 [2.05, 13.62] n=27 | 11.44 [4.78, 27.85] n=80 | 0.014 | 0.033 |
| IL19 | 2.50 [2.30, 3.40] n=16 | 19.00 [18.00, 20.00] n=54 | <0.001 | <0.001 |
| IL26 | 1.70 [1.60, 1.70] n=16 | 33.00 [9.52, 48.00] n=54 | <0.001 | <0.001 |
| IL27 | 3.75 [3.20, 6.50] n=16 | 65.00 [65.00, 65.00] n=54 | <0.001 | <0.001 |
| IL32 | 9.65 [8.32, 20.60] n=16 | 47.98 [14.51, 87.00] n=47 | 0.002 | 0.006 |
| IL34 | 14.85 [12.57, 47.58] n=16 | 44.30 [25.62, 54.30] n=54 | 0.037 | 0.071 |
| IL35 | 16.82 [5.87, 60.05] n=16 | 45.00 [43.00, 103.84] n=54 | 0.016 | 0.037 |
| CCL2 | 28.61 [17.64, 30.00] n=27 | 31.18 [19.40, 58.85] n=80 | 0.049 | 0.108 |
| CCL27 | 62.93 [44.77, 116.15] n=27 | 46.65 [27.15, 64.05] n=80 | 0.003 | 0.013 |
| CXCL1 | 30.22 [20.90, 43.92] n=27 | 23.23 [13.71, 30.19] n=80 | 0.022 | 0.061 |
| CXCL9 | 157.22 [55.02, 281.44] n=27 | 1890.86 [893.72, 2904.33] n=80 | <0.001 | <0.001 |
| CXCL10 | 128.46 [58.28, 339.23] n=27 | 520.53 [212.08, 1323.28] n=80 | <0.001 | <0.001 |
| CHI3L1 | 15330.14 [9583.69, 19510.30] n=16 | 22313.71 [16627.44, 27183.99] n=54 | 0.002 | 0.005 |
| IFNA2 | 9.85 [7.64, 14.13] n=27 | 21.59 [16.46, 32.58] n=54 | <0.001 | <0.001 |
| IFNγ | 11.41 [6.89, 20.74] n=26 | 57.13 [29.48, 110.47] n=79 | <0.001 | <0.001 |
| IFNL1 | 6.66 [2.70, 17.79] n=16 | 38.19 [23.00, 39.40] n=54 | <0.001 | <0.001 |
| IFNL2 | 21.91 [2.30, 37.95] n=16 | 43.00 [28.45, 53.89] n=54 | 0.010 | 0.013 |
| LIF | 2.50 [1.33, 6.00] n=27 | 5.45 [2.65, 11.00] n=80 | 0.002 | 0.005 |
| NGF | 1.57 [0.68, 3.00] n=27 | 4.00 [1.95, 4.00] n=80 | <0.001 | <0.001 |
| CSF1 | 2.50 [1.39, 4.00] n=27 | 7.00 [2.41, 7.00] n=80 | <0.001 | 0.002 |
| MMP1 | 42.72 [12.20, 71.78] n=27 | 129.84 [55.69, 213.90] n=78 | <0.001 | <0.001 |
| MMP2 | 94.22 [30.96, 215.47] n=27 | 267.78 [191.88, 415.22] n=75 | <0.001 | <0.001 |
| MMP3 | 353.78 [126.29, 794.66] n=27 | 958.12 [597.85, 1258.90] n=78 | <0.001 | <0.001 |
| MMP7 | 48.67 [19.23, 251.22] n=27 | 666.76 [84.31, 1771.39] n=80 | <0.001 | <0.001 |
| MMP8 | 23.76 [15.33, 39.88] n=27 | 88.57 [44.99, 304.87] n=80 | <0.001 | <0.001 |
| MMP9 | 621.09 [176.28, 2073.84] n=27 | 5724.90 [1526.57, 18605.87] n=80 | <0.001 | <0.001 |
| MMP10 | 32.30 [22.30, 76.34] n=27 | 217.54 [98.74, 311.69] n=80 | <0.001 | <0.001 |
| MMP13 | 28.60 [11.00, 53.78] n=27 | 129.97 [68.45, 268.98] n=80 | <0.001 | <0.001 |
| MMP12 | 144.04 [25.43, 399.38] n=27 | 445.42 [210.74, 704.51] n=78 | 0.006 | 0.006 |
| BGLAP | 779.60 [636.87, 939.98] n=16 | 519.99 [317.18, 844.38] n=54 | 0.041 | 0.059 |
| SPP1 | 8043.93 [4764.56, 11907.09] n=16 | 14008.81 [7604.72, 23790.53] n=54 | 0.010 | 0.018 |
| PTX3 | 146.88 [19.44, 239.96] n=16 | 282.62 [160.35, 424.28] n=54 | 0.003 | 0.005 |
| SCF | 29.95 [26.16, 42.89] n=27 | 44.45 [31.64, 63.15] n=80 | 0.004 | 0.008 |
| CLEC11A | 1085.91 [505.27, 2063.39] n=27 | 5283.13 [3376.36, 9283.41] n=80 | <0.001 | <0.001 |
| sTNFRSF1A | 899.08 [365.00, 1419.22] n=16 | 2571.62 [1900.13, 3723.82] n=54 | <0.001 | <0.001 |
| sTNFRSF1B | 4880.41 [1838.82, 7583.45] n=16 | 16839.75 [11993.60, 20623.79] n=54 | <0.001 | <0.001 |
| sCD163 | 361.24 [298.61, 663.13] n=16 | 1181.97 [712.99, 1661.14] n=54 | <0.001 | <0.001 |
| sIL6R | 5634.20 [2359.65, 10338.55] n=27 | 14394.67 [10550.03, 18985.69] n=80 | <0.001 | <0.001 |
| TNFRSF8 | 432.18 [318.78, 467.40] n=16 | 1641.16 [1183.28, 3111.51] n=54 | <0.001 | <0.001 |
| TNFSF14 | 7.40 [5.55, 8.60] n=16 | 8.60 [8.40, 9.40] n=54 | 0.001 | 0.002 |
| LTA | 0.50 [0.39, 0.65] n=27 | 0.38 [0.23, 0.63] n=80 | 0.019 | 0.037 |
| TSLP | 17.90 [12.86, 23.94] n=16 | 11.59 [7.04, 18.63] n=54 | 0.023 | 0.037 |

Comparison was conducted using Mann–Whitney U test.

Table 3. Serum cytokine, chemokine and MMP levels in functional CCR5/*Δ32CCR5* heterozygous NE cases and controls (median [Q1, Q3]).

| Variable (pg/ml) | Control | CCR5 heterozygous | p | Adjusted p |
| --- | --- | --- | --- | --- |
| IL1α | 0.80 [0.18, 1.33] n=27 | 0.15 [0.11, 0.21] n=16 | 0.002 | 0.009 |
| IL2RA | 27.52 [13.53, 40.51] n=27 | 97.53 [68.54, 225.64] n=16 | <0.001 | <0.001 |
| IL3 | 67.64 [33.36, 101.21] n=27 | 177.04 [83.59, 203.88] n=16 | 0.003 | 0.009 |
| IL8 | 11.40 [8.60, 32.58] n=25 | 63.22 [35.25, 163.92] n=6 | 0.007 | 0.018 |
| IL10 | 6.60 [3.52, 10.45] n=27 | 21.25 [7.14, 40.51] n=16 | 0.002 | 0.009 |
| IL11 | 1.49 [0.87, 2.27] n=27 | 3.22 [1.99, 13.14] n=6 | 0.047 | 0.105 |
| IL12B | 70.80 [36.59, 154.07] n=27 | 214.68 [124.50, 273.19] n=16 | 0.002 | 0.009 |
| IL16 | 105.65 [72.40, 141.87] n=27 | 135.59 [113.27, 186.78] n=16 | 0.022 | 0.054 |
| IL18 | 5.76 [2.05, 13.62] n=27 | 21.43 [6.72, 30.76] n=16 | 0.006 | 0.017 |
| IL19 | 2.50 [2.30, 3.40] n=16 | 19.50 [19.00, 20.75] n=6 | 0.002 | 0.009 |
| IL26 | 1.70 [1.60, 1.70] n=16 | 48.00 [36.00, 48.00] n=6 | 0.002 | 0.009 |
| IL27 | 3.75 [3.20, 6.50] n=16 | 65.00 [65.00, 65.00] n=6 | 0.002 | 0.009 |
| IL32 | 9.65 [8.32, 20.60] n=16 | 92.00 [92.00, 92.00] n=5 | 0.004 | 0.012 |
| CCL27 | 62.93 [44.77, 116.15] n=27 | 33.77 [28.16, 49.84] n=16 | 0.001 | 0.005 |
| CXCL9 | 157.22 [55.02, 281.44] n=27 | 1751.24 [1106.26, 3705.88] n=16 | <0.001 | <0.001 |
| CXCL10 | 128.46 [58.28, 339.23] n=27 | 568.92 [133.20, 1806.06] n=16 | 0.008 | 0.031 |
| CSF2 | 7.31 [4.73, 13.66] n=27 | 1.93 [1.20, 7.23] n=16 | 0.026 | 0.113 |
| IFNβ | 11.41 [6.89, 20.74] n=26 | 63.15 [45.23, 114.03] n=16 | <0.001 | <0.001 |
| IFNL1 | 6.66 [2.70, 17.79] n=16 | 35.66 [25.23, 39.40] n=6 | 0.014 | 0.036 |
| LIF | 2.50 [1.33, 6.00] n=27 | 6.56 [4.36, 11.00] n=16 | 0.035 | 0.113 |
| CSF1 | 2.50 [1.39, 4.00] n=27 | 5.07 [3.75, 7.00] n=16 | 0.007 | 0.092 |
| MMP1 | 42.72 [12.20, 71.78] n=27 | 103.39 [35.97, 149.97] n=16 | 0.047 | 0.106 |
| MMP2 | 94.22 [30.96, 215.47] n=27 | 20.75 [11.62, 46.52] n=16 | 0.001 | 0.005 |
| MMP7 | 48.67 [19.23, 251.22] n=27 | 558.12 [120.58, 1294.25] n=16 | 0.002 | 0.008 |
| MMP9 | 621.09 [176.28, 2073.84] n=27 | 1680.99 [1117.12, 2630.14] n=16 | 0.042 | 0.106 |
| NGF | 1.57 [0.68, 3.00] n=27 | 2.38 [1.99, 4.00] n=16 | 0.022 | 0.052 |
| SCF | 29.95 [26.16, 42.89] n=27 | 67.32 [42.44, 81.81] n=16 | 0.001 | 0.002 |
| CLEC11A | 1085.91 [505.27, 2063.39] n=27 | 6385.62 [2932.68, 9803.79] n=16 | <0.001 | <0.001 |
| sIL6Ra | 5634.20 [2359.65, 10338.55] n=27 | 15046.73 [13793.24, 16907.83] n=6 | 0.045 | 0.116 |
| sTNFRSF1A | 899.08 [365.00, 1419.22] n=16 | 2232.00 [1548.48, 3186.55] n=6 | 0.027 | 0.163 |
| sTNFRSF1B | 4880.41 [1838.82, 7583.45] n=16 | 16658.56 [11431.52, 18617.04] n=6 | 0.033 | 0.163 |
| TSLP | 17.90 [12.86, 23.94] n=16 | 8.48 [3.67, 13.45] n=6 | 0.015 | 0.097 |

Comparison was conducted using Mann–Whitney U test.

Table 4. Serum cytokine, chemokine and MMP levels in functional CCR5 homozygous and functional CCR5/*Δ32CCR5* heterozygous NE cases (median [Q1, Q3]).

| Variable (pg/ml) | functional *CCR5*/*Δ32CCR5* heterozygous | functional *CCR5* homozygous | p | Adjusted p |
| --- | --- | --- | --- | --- |
| IL9 | 14.26 [8.93, 30.39] n=16 | 36.89 [15.37, 52.39] n=79 | 0.011 | 0.203 |
| IL32 | 92.00 [92.00, 92.00] n=5 | 47.98 [14.51, 87.00] n=47 | 0.014 | 0.203 |
| MMP2 | 20.75 [11.62, 46.52] n=16 | 267.78 [191.88, 415.22] n=75 | <0.001 | <0.001 |
| MMP3 | 408.73 [317.94, 530.58] n=16 | 958.12 [597.85, 1258.90] n=78 | <0.001 | <0.001 |
| MMP8 | 14.47 [7.32, 31.16] n=16 | 88.57 [44.99, 304.87] n=80 | <0.001 | <0.001 |
| MMP9 | 1680.99 [1117.12, 2630.14] n=16 | 5724.90 [1526.57, 18605.87] n=80 | 0.036 | 0.047 |
| MMP10 | 20.15 [10.47, 51.18] n=16 | 217.54 [98.74, 311.69] n=80 | <0.001 | <0.001 |
| MMP13 | 42.74 [27.89, 54.04] n=16 | 129.97 [68.45, 268.98] n=80 | <0.001 | <0.001 |
| MMP12 | 121.09 [59.00, 177.79] n=16 | 445.42 [210.74, 704.51] n=78 | <0.001 | <0.001 |

Comparison was conducted using Mann–Whitney U test.

Table 5. Severity-associated analytes

| Analyte | Mild form | Severe form | p | Pseudomedian | CI(L) | CI(U) | CLES | RBSC | Adjusted p |
| --- | --- | --- | --- | --- | --- | --- | --- | --- | --- |
| Creatinine | 122.00 [102.50, 173.50] n=43 | 145.00 [128.00, 281.00] n=31 | 0.032 | -28.00 | -55.00 | -3.00 | 0.64 | 0.29 | 0.129 |
| MMP2 | 154.59 [34.29, 340.01] n=41 | 249.39 [193.17, 375.46] n=29 | 0.038 | -113.19 | -180.61 | -2.12 | 0.64 | 0.28 | 0.171 |
| MMP10 | 106.86 [33.38, 257.02] n=43 | 242.91 [106.86, 423.89] n=31 | 0.012 | -95.87 | -195.06 | -16.34 | 0.67 | 0.34 | 0.112 |
| LTA | 0.44 [0.24, 0.76] n=43 | 0.24 [0.18, 0.35] n=31 | 0.004 | 0.15 | 0.05 | 0.34 | 0.28 | -0.44 | 0.040 |
| TRAIL | 30.41 [16.14, 69.17] n=43 | 21.04 [10.33, 37.33] n=31 | 0.045 | 11.60 | 0.00 | 25.21 | 0.36 | -0.28 | 0.227 |
| CSF2 | 2.81 [1.42, 8.55] n=43 | 8.70 [4.30, 13.79] n=31 | 0.013 | -3.39 | -6.62 | -0.65 | 0.67 | 0.34 | 0.170 |
| HGF | 161.31 [25.41, 384.25] n=43 | 34.38 [14.48, 139.54] n=31 | 0.012 | 75.26 | 6.79 | 167.93 | 0.33 | -0.35 | 0.028 |
| NGF | 4.00 [1.45, 4.00] n=43 | 4.00 [4.00, 4.00] n=31 | 0.000 | -1.00 | -2.05 | 0.00 | 0.55 | 0.10 | 0.000 |
| PDGFBB | 549.09 [282.86, 1194.07] n=43 | 264.66 [186.88, 449.47] n=31 | 0.007 | 233.29 | 59.41 | 436.64 | 0.32 | -0.37 | 0.024 |
| IFNγ | 67.84 [46.23, 134.97] n=43 | 41.37 [13.97, 61.10] n=30 | 0.002 | 33.74 | 13.77 | 57.42 | 0.28 | -0.43 | 0.011 |
| IL4 | 2.72 [1.80, 4.87] n=43 | 4.25 [3.08, 4.82] n=31 | 0.046 | -1.00 | -1.82 | 0.00 | 0.63 | 0.27 | 0.295 |
| IL32 | 87.00 [45.97, 92.00] n=43 | 36.15 [14.44, 87.00] n=26 | 0.035 | 16.66 | 0.00 | 68.07 | 0.25 | -0.51 | 0.295 |
| CCL27 | 42.73 [28.16, 58.41] n=43 | 60.03 [44.53, 81.38] n=31 | 0.002 | -18.91 | -30.03 | -7.71 | 0.71 | 0.42 | 0.011 |
| CXCL12 | 41.49 [21.67, 61.50] n=43 | 61.50 [44.22, 67.40] n=31 | 0.001 | -19.38 | -30.75 | -7.57 | 0.71 | 0.42 | 0.011 |

Continuous variables are presented with their respective median, first (Q1) and third (Q3) quartiles (median [Q1, Q3], total n). Comparison was conducted using Mann–Whitney U test. CI(L) and CI(U) – lower and upper margins of nonparametric 95% confidence interval for pseudomedian. CLES - common language effect size. RBSC - rank-biserial correlation (the simple difference formula).

Table 6. Pairwise spearman correlation analysis to remove redundant features

| Analyte 1 | Analyte 2 | Number of complete observations | Correlation coefficient | p |
| --- | --- | --- | --- | --- |
| Creatinine | MMP2 | 70 | 0.01 | 0.963 |
| Creatinine | MMP10 | 74 | 0.11 | 0.347 |
| Creatinine | LTA | 74 | 0.01 | 0.954 |
| Creatinine | TRAIL | 74 | 0.01 | 0.920 |
| Creatinine | CSF2 | 74 | 0.32 | 0.005 |
| Creatinine | HGF | 74 | 0.08 | 0.486 |
| Creatinine | NGF | 74 | 0.13 | 0.265 |
| Creatinine | PDGFBB | 74 | 0.17 | 0.137 |
| Creatinine | IFNγ | 73 | 0.22 | 0.059 |
| Creatinine | IL4 | 74 | 0.30 | 0.009 |
| Creatinine | IL32 | 46 | 0.02 | 0.903 |
| Creatinine | CCL27 | 74 | 0.14 | 0.237 |
| Creatinine | CXCL12 | 74 | 0.08 | 0.516 |
| MMP2 | MMP10 | 70 | 0.82 | <0.001 |
| MMP2 | LTA | 70 | -0.12 | 0.322 |
| MMP2 | TRAIL | 70 | -0.03 | 0.785 |
| MMP2 | CSF2 | 70 | 0.20 | 0.089 |
| MMP2 | HGF | 70 | 0.00 | 0.978 |
| MMP2 | NGF | 70 | 0.37 | 0.002 |
| MMP2 | PDGFBB | 70 | -0.07 | 0.574 |
| MMP2 | IFNγ | 69 | 0.00 | 0.980 |
| MMP2 | IL4 | 70 | 0.18 | 0.130 |
| MMP2 | IL32 | 44 | -0.28 | 0.066 |
| MMP2 | CCL27 | 70 | 0.23 | 0.054 |
| MMP2 | CXCL12 | 70 | 0.35 | 0.003 |
| MMP10 | LTA | 74 | -0.09 | 0.424 |
| MMP10 | TRAIL | 74 | -0.02 | 0.849 |
| MMP10 | CSF2 | 74 | 0.22 | 0.065 |
| MMP10 | HGF | 74 | -0.02 | 0.893 |
| MMP10 | NGF | 74 | 0.41 | <0.001 |
| MMP10 | PDGFBB | 74 | -0.02 | 0.852 |
| MMP10 | IFNγ | 73 | -0.19 | 0.111 |
| MMP10 | IL4 | 74 | 0.18 | 0.131 |
| MMP10 | IL32 | 46 | -0.23 | 0.118 |
| MMP10 | CCL27 | 74 | 0.28 | 0.016 |
| MMP10 | CXCL12 | 74 | 0.32 | 0.005 |
| LTA | TRAIL | 74 | 0.52 | <0.001 |
| LTA | CSF2 | 74 | -0.35 | 0.002 |
| LTA | HGF | 74 | 0.63 | <0.001 |
| LTA | NGF | 74 | -0.37 | 0.001 |
| LTA | PDGFBB | 74 | 0.42 | <0.001 |
| LTA | IFNγ | 73 | 0.23 | 0.048 |
| LTA | IL4 | 74 | -0.45 | <0.001 |
| LTA | IL32 | 46 | -0.15 | 0.306 |
| LTA | CCL27 | 74 | -0.36 | 0.002 |
| LTA | CXCL12 | 74 | -0.29 | 0.011 |
| TRAIL | CSF2 | 74 | -0.25 | 0.034 |
| TRAIL | HGF | 74 | 0.66 | <0.001 |
| TRAIL | NGF | 74 | -0.32 | 0.005 |
| TRAIL | PDGFBB | 74 | 0.49 | <0.001 |
| TRAIL | IFNγ | 73 | 0.25 | 0.031 |
| TRAIL | IL4 | 74 | -0.24 | 0.038 |
| TRAIL | IL32 | 46 | -0.13 | 0.376 |
| TRAIL | CCL27 | 74 | -0.16 | 0.166 |
| TRAIL | CXCL12 | 74 | -0.22 | 0.055 |
| CSF2 | HGF | 74 | -0.26 | 0.025 |
| CSF2 | NGF | 74 | 0.50 | <0.001 |
| CSF2 | PDGFBB | 74 | -0.18 | 0.123 |
| CSF2 | IFNγ | 73 | 0.20 | 0.092 |
| CSF2 | IL4 | 74 | 0.56 | <0.001 |
| CSF2 | IL32 | 46 | -0.04 | 0.778 |
| CSF2 | CCL27 | 74 | 0.21 | 0.074 |
| CSF2 | CXCL12 | 74 | 0.49 | <0.001 |
| HGF | NGF | 74 | -0.37 | 0.001 |
| HGF | PDGFBB | 74 | 0.60 | <0.001 |
| HGF | IFNγ | 73 | 0.23 | 0.046 |
| HGF | IL4 | 74 | -0.11 | 0.357 |
| HGF | IL32 | 46 | -0.23 | 0.122 |
| HGF | CCL27 | 74 | -0.11 | 0.360 |
| HGF | CXCL12 | 74 | -0.15 | 0.213 |
| NGF | PDGFBB | 74 | -0.40 | <0.001 |
| NGF | IFNγ | 73 | -0.19 | 0.108 |
| NGF | IL4 | 74 | 0.36 | 0.002 |
| NGF | IL32 | 46 | -0.28 | 0.064 |
| NGF | CCL27 | 74 | 0.39 | 0.001 |
| NGF | CXCL12 | 74 | 0.57 | <0.001 |
| PDGFBB | IFNγ | 73 | 0.22 | 0.059 |
| PDGFBB | IL4 | 74 | -0.03 | 0.787 |
| PDGFBB | IL32 | 46 | -0.15 | 0.329 |
| PDGFBB | CCL27 | 74 | -0.12 | 0.309 |
| PDGFBB | CXCL12 | 74 | -0.28 | 0.015 |
| IFNγ | IL4 | 73 | 0.28 | 0.018 |
| IFNγ | IL32 | 45 | -0.14 | 0.368 |
| IFNγ | CCL27 | 73 | -0.09 | 0.436 |
| IFNγ | CXCL12 | 73 | -0.10 | 0.402 |
| IL4 | IL32 | 46 | 0.03 | 0.861 |
| IL4 | CCL27 | 74 | 0.34 | 0.004 |
| IL4 | CXCL12 | 74 | 0.47 | <0.001 |
| IL32 | CCL27 | 46 | -0.07 | 0.643 |
| IL32 | CXCL12 | 46 | 0.18 | 0.242 |
| CCL27 | CXCL12 | 74 | 0.39 | 0.001 |

If two variables had absolute value of correlation coefficient higher than 0.5, the mean absolute correlation of each variable was calculated. Then the variable with the largest mean absolute correlation was removed.

Figure 1. Relevance of severity-associated continuous variables reported as common language effect size (using package “canprot”). In order to retain only the most prognostically valuable analytes of those identified, redundant features were removed using pairwise spearman correlation analysis with packages: “caret” and “reshape2”. If two variables had absolute value of correlation coefficient higher than 0.5, the mean absolute correlation of each variable was calculated (only correlation coefficients significantly different from zero were considered). Then the variable with the largest mean absolute correlation was removed.

Figure 2. Relevance of severity-associated continuous variables reported as pseudomedian and its nonparametric 95% confidence interval (point range in the middle). Boxes represent third, second and first quartiles (top to bottom), while whiskers indicate maximum and minimum levels of a given analyte in corresponding group. In order to retain only the most prognostically valuable analytes of those identified, redundant features were removed using pairwise spearman correlation analysis with packages: “caret” and “reshape2”. If two variables had absolute value of correlation coefficient higher than 0.5, the mean absolute correlation of each variable was calculated (only correlation coefficients significantly different from zero were considered). Then the variable with the largest mean absolute correlation was removed.
